# Effect of Different Dietary Regimes on the Gut Microbiota and Fecal Metabolites of Père David’s Deer

**DOI:** 10.3390/ani12050584

**Published:** 2022-02-25

**Authors:** Junai Zhen, Yijun Ren, Huidan Zhang, Xueli Yuan, Libo Wang, Hua Shen, Ping Liu, Yuqing Chen

**Affiliations:** 1 Jiangsu Province Key Laboratory for Molecular and Medical Biotechnology, Life Sciences College, Nanjing Normal University, Nanjing 210000, China; 191202090@njnu.edu.cn (J.Z.); 201201029@njnu.edu.cn (H.Z.); 201202042@njnu.edu.cn (X.Y.); liuping0805@njnu.edu.cn (P.L.); 2 Jiangsu Dafeng Père David’s Deer National Nature Reserve, Yancheng 224000, China; ryijun2017@163.com (Y.R.); dfsh168@126.com (H.S.); wanglibo2008@126.com (L.W.)

**Keywords:** silage, combination diet, metabolic pathway, nutrition

## Abstract

**Simple Summary:**

Père David’s deer is native to the middle and lower reaches of the Yangtze River and the Yellow River in China. However, the wild population became extinct in China around 1900. In 1986, 39 Père David’s deer were reintroduced into Dafeng. Up until now, its wild population has reached 2658, with a total of 6119 in 2021. At present, due to the continuous increase in the population, the repeated grazing on the same plants by the Père David’s deer has affected the re-growth of plants, which has led to insufficient natural food. Therefore, feeding supplement with silage is necessary. As a key nutritional factor, diet is the most important for the gut microbiota and metabolites of wild animals. In order to determine the effect of different dietary patterns on the nutrition and health of Père David’s deer in Dafeng Reserve in spring, we conducted a comprehensive analysis of Père David’s deer feces by UPLC-MS/MS and 16S rRNA gene sequencing to reveal its intestinal chemical environment and the differences in the fecal microbiome. Altogether, our data explored the significant changes in the gut microbiota and metabolic pathways during the transition from full silage to a combination diet with silage and plant in spring. These data provided important information to make more reasonable measures for Père David’s deer’s protection.

**Abstract:**

A deep understanding of the effect of seasonal dietary changes on the nutrition and health of Père David’s deer in Dafeng Reserve will contribute greatly to Père David’s deer’s protection. In this reserve, there were three seasonal dietary regimes: feeding on naturally occurring plants (PLANT diet), silage (SILAGE diet), and a combination of natural plants and silage (COMB diet). To some extent, the COMB diet reflects the seasonal transition from silage to the all-natural plant diet, especially in early spring. However, little is known regarding the gut microbiota changes and metabolic consequences under the COMB diet. Based on 16S rRNA sequencing and ultra-high performance liquid chromatography combined with tandem mass spectrometry, the gut microbiota and fecal metabolites of Père David’s deer under these three diets were compared. Results showed the alpha diversity of the gut microbiota was significantly lower under the COMB diet compared to either the SILAGE or PLANT diets. Although no significant changes were observed in the core phyla, Firmicutes and Bacteroidetes, among the three dietary regimes, a significant lower abundance of several other phyla (Spirochaetes, Melainabacteria, Proteobacteria, and Verrucobacteria) was observed in the COMB diet compared to the SILAGE diet. A greater number of fecal metabolite differences was identified between the COMB and SILAGE or COMB and PLANT diets than between the SILAGE and PLANT diets, suggesting that the COMB diet had more of an effect on the metabolism of Père David’s deer. The integrated pathway analysis showed that several metabolic pathways were significantly affected by the different dietary regimes, such as tryptophan metabolism, vitamin metabolism, and the platelet activation pathways. These metabolic changes reflect the responses and adaptations of Père David’s deer to different diets. Taken overall, our data reveal the difference in the gut microbiota and metabolic pathways of Père David’s deer under three dietary regimes in Dafeng Reserve, which provides important information for Père David’s deer conservation.

## 1. Introduction

Père David’s deer (*Elaphurus davidianus*) are native to the middle and lower reaches of the Yangtze and Yellow Rivers in China. Fossil data showed that Père David’s deer originated at least two million years ago and were most numerous between approximately 10,000 and 3000 years ago [1]. However, the wild population became extinct in China around 1900 due to human activity, climate change, and other unknown factors. Fortunately, before the wild population extinction in China, captive populations were established overseas and formed the basis for a reintroduction to China in 1985 [2]. Currently, three large native populations have been established in Dafeng (Jiangsu Province), Shishou (Hubei Province), and Dongting Lake (Hunan Province), as well as nearly 100 other small populations distributed throughout China [3]. Dafeng Reserve is located on the coast of the Yellow Sea in eastern China. In 1986, 39 Père David’s deer were reintroduced to Dafeng. After a gradual adaptation to the local ecological environment and photoperiod, the population reached 354 in 1998, and then a new project was established to release more semi-free Père David’s deer into the wild in Dafeng to fully restore the original wild population. Since 2003, the wild population has gradually recovered on the coastal beach area, and reached 2658 in 2021. The latest statistics show that there were actually 6119 individuals of the semi-free population and wild population in 2021, an approximately 174-fold increase in the population since the first reintroduction to the Dafeng Reserve (http://www.chinamlw.org/, accessed on 19 June 2021). These increases show that the restoration is a very successful example of ex situ conservation. 

The living area of Père David’s deer in Dafeng Reserve is composed of three core areas: core areas I (DFI), core areas II (DFII), and core areas (DFIII). DFI and DFII are the semi-free areas (6.7 km^2^), and DFIII (10.05 km^2^) is the wild area. Deer in the semi-free areas are fenced-in and eat naturally occurring plants. However, the continuous increase in the population, its high density, and the limited available habitat, repeated grazing on the same plants, and trampling of the vegetation by the deer have limited the amount of plant regrowth [4]. Additionally, due to seasonal variation in the climate of the reserve, the natural vegetation withers from November to April, leading to a shortage of natural food. Furthermore, the saline–alkaline land along the coast in the semi-free and wild areas is unsuitable for their natural food, greatly reducing the variety of edible plant species. Therefore, supplementary feeding with silage has become important and necessary for the survival of Père David’s deer in Dafeng, especially in winter [5]. This results in the deer utilizing three different dietary regimes during the year: natural occurring plants, silage, and a combination of natural plants and silage. Usually, most deer eat silage in winter, and, in spring, some eat silage together with natural plants. A previous report showed that the diet determined the key nutritional parameters for wildlife survival [6]. However, to date, few studies have investigated the effect of dietary changes on the nutrition and health of Père David’s deer, especially the combination diet with silage and natural plants. Therefore, it is necessary to deeply understand the differences in digestion and nutrition under different diets, so as to plan better conservation measures.

Many studies have shown large, complex microbial communities in the gastrointestinal tracts of wild animals, which are essential in maintaining the hosts’ health [7]. Further analyses have revealed that differences in the diet can affect the composition and size of the gut microbial community in both captive and wild animals [8,9,10]. Although the ruminal microbiome plays an important role in the nutrition and digestion for Ruminantia, the subsequent process by the gut microbiome leads to the complete digestion and utilization of food [11]. Thus, the gut microbiome is very important in maintaining the nutrition and health of ruminants. A comparison of Père David’s deer from Beijing and Shisou revealed that their food sources may change during ex situ conservation activities, resulting in differences in the gut microbiota [12]. Wang et al. suggested that the gut microorganisms of Père David’s deer in Dafeng had potentially co-evolved with their diet, reflecting a local adaptation of the translocated populations to their new environments [13]. A recent study revealed that there were clear differences in the gut microbial community structure between captive and wild Père David’s deer in Dafeng Reserve [5]. Currently, no information is available relating to the relationships among the diet, gut microbiota, and metabolic consequences, especially in the COMB diet occurring during seasonal dietary transitions.

Feces are important source of samples for most gut microbiota studies [14]. However, there is a bias in the estimation of gut microbiota with feces because the fecal microbiota does not fully represent that of the gastrointestinal tract [15]. Due to their convenience, non-invasiveness, and sufficient biomass for analysis, feces are still the major source of samples for gut microbiota studies, especially in wild animals [16,17]. Changes in gut microbiota could cause differences in metabolic phenotypes [18]. Currently, non-targeted metabolomics has been used to reveal such changes. In this study, we performed a comprehensive analysis of the metabolites using ultra-high performance liquid chromatography combined with a tandem mass spectrometry (UPLC-MS/MS) analysis of the feces of Père David’s deer under three dietary regimes to reveal their gut chemical environments. We also analyzed the differences in feces microbiomes based on 16S rRNA gene sequencing of individuals with different dietary regimes.

## 2. Materials and Methods

### 2.1. Diet Survey and Analysis

Silage for Père David’s deer in Dafeng Reserve was full-plant corn silage (corns are crushed, sealed, and fermented) supplemented with soybean meal and bran. The silage samples were weighed and dried at 60 °C. A total of 200 g of dry samples were crushed with a pulverizer. The chemical composition (including crude protein, non-fiber carbohydrates, neutral detergent fiber, acid detergent fiber, fat, ash, lactic acid, acetic acid, and butanoic acid) were analyzed by near infrared spectroscopy (NIR). Measurements were carried in the range from 400 to 2500 nm. Four batches of silage samples were detected. This work was conducted by the King Techina Company (Hangzhou, China) using the silage analyzer NIRS™ DS2500 F (FOSS Electric A/S, Hillerød, Denmark). The naturally occurring plants preferred by Père David’s deer in Dafeng in spring were as previously described, mainly including *Pennisetum alopecuroides*, *Spartina alterniflora*, *Imperata cylindrica*, and *Phragmites communis* [19]. 

### 2.2. Fecal Sample Collection 

Fecal samples of Père David’s deer individuals were obtained from Dafeng Reserve (33°05′ N, 120°49′ E), located in East China on the shore of the Yellow Sea. The climate is characterized as obvious transitional, oceanic, and monsoonal, with an annual average temperature of 14.1 °C. January is the coldest month, with a monthly average temperature of 0.8 °C, and July is the hottest, with a monthly average temperature of 27 °C [20]. From March to June in 2020, nearly 60 fresh fecal samples from different individuals (approximately 4 to 12 years old) were collected. No extreme weather occurred during this period. No antibiotics or other medications were used in the sampling areas. These samples were collected by 3 batches (March, April, June), with each batch (approximately 20 samples) being collected simultaneously in different areas (DFI, DFII, and DFIII) at the same day from 6 a.m. to 7 a.m. The numbers in each areas were considered as follows. Briefly, fecal samples from the SILAGE diet were mainly collected from DFII, which was very sparsely vegetated. The fecal samples from the COMB diet (mixed natural vegetation and silage) were mainly collected from DFI, where there are insufficient naturally occurring plants and the deer diets are supplemented with silage. The PLANT diet (natural vegetation) samples were collected from DFIII, which is covered by several plant species. In addition, the texture of the feces was also considered. The feces of deer that were more likely to eat naturally occurring plants were usually soft and full of fiber from plants, whereas, if the feces were hard and uniformly fine inside, the corresponding individual was more likely to be eating silage. In order to ensure the freshness of the samples, they were collected aseptically immediately after defecation. In order to eliminate the influence of other factors, the inner part of the feces not exposed to the air were removed and stored in sterile tubes. The sterile tubes with samples were quickly placed into dry ice for storage and transportation and then stored at −80 °C for further analysis. We did not introduce any substances that could interfere with the habitat of Père David’s deer. The study complied with both the agreements made with the China Wildlife Conservation Association and with Chinese law.

### 2.3. Microscopic Examination and Identification

To ensure consistency of fecal samples with dietary patterns, microscopic observation was conducted. Briefly, silage and all of the fecal samples were dried for 24 h at 60 °C, crushed with a pulverizer, and then filtered with a 60-mesh and then a 100-mesh sieve to collect the fine particles. Sodium hypochlorite solution was then added to the fine particles and stirred every 1 h. After 4 h, the samples were filtered through a 200-mesh sieve and rinsed with distilled water to remove the sodium hypochlorite residue. Finally, the samples were placed on a glass slide and observed with a light microscope (Leica, Wetzlar, Germany) using LAS software (v4.7).

### 2.4. 16. S rRNA Microbial Community Analysis

The total DNA was extracted from a core part of the frozen fecal sample using a Magnetic Soil and Stool DNA Kit (TIANGEN, Beijing, China). Then, a NanoDrop 2000 UV-vis spectrophotometer was used to detect the DNA concentration and 1 ng/μL DNA was used for follow-up analysis. The V3-V4 regions of the 16S rRNA gene were amplified using forward and reverse primers: 341F (5′-CCTAYGGGRBGCASCAG-3′) and 806R (5′-GGACTACNNGGGTATCTAAT-3’), respectively [21]. PCR amplification was performed using Phusion^®^ High-Fidelity PCR Master Mix with GC Buffer (New England Biolabs, Ipswich, MA, USA). The PCR products were purified using a QIAquick Gel Extraction Kit (Qiagen, Valencia, CA, USA) and quantified on a NanoDrop 2000 UV-vis spectrophotometer (Thermo Scientific, USA). The sequencing library was then prepared using a TruSeq^®^ DNA PCR-Free Sample Preparation Kit (Illumina Inc., San Diego, CA, USA), and a NovaSeq6000 system was used for sequencing. Using FLASH (v1.2.7) to splice the reads of each sample, the spliced sequences were obtained and used as the raw data. 

### 2.5. Sequence Processing and Analysis

Use QIIME (V1.9.1) for quality control and refer to the following criteria: (1) cut raw tags from the first low-quality base site with consecutive low-quality values (the default quality threshold is ≤19), and the number of bases reaches the set length (the default length is 3); (2) further filter out the tags whose continuous high-quality base length is less than 75% of the tag length. Use Uparse software with 97% identity to cluster all effective tags into OTUs [22]. Thus, based on 13,236,835 high-quality reads obtained, the species annotation analysis was performed using Mothur method and SSUrRNA database of SILVA132 (set threshold 0.8–1) to obtain the taxonomic information and statistics of the community composition of each sample at each taxonomic level: kingdom, phylum, class, order, family, and genus [23]. The alpha diversity indices were calculated at the OTU level (including the Chao1 indices and Shannon index). The Beta diversity analysis, including principal component analysis (PCA) and principal co-ordinate analysis (PCoA) analysis, was calculated and visualized using R packages. 

### 2.6. UPLC-MS/MS Analysis

A range of small molecules (most < 1000 Da) were detected in the fecal samples using untargeted metabolomics analysis performed using UPLC-MS/MS (QTRAP^®^) equipped with an electrospray ionization (ESI) source in positive and negative ion modes with minor modification. Integration of the areas under the extracted ion chromatographic peaks of all metabolites and integral correction of the chromatographic peaks of the same metabolite in different samples were performed, as described previously [24]. Briefly, samples (50 mg per sample) were pretreated and then separated using a UPLC system, followed by mass spectrometry analysis. Samples were mixed in equal amounts to prepare quality control (QC) samples, which were evenly spaced between the injections. The original data were imported into the metabolomics processing software Analyst v1.6.3, and then the MS and MS/MS data were matched with the metabolite database MWDB (Metware Biotechnology) to identify the metabolites. 

### 2.7. Metabolomic Analysis

PCA of the samples (including the QC samples) was conducted among the groups of samples, and for the degree of variability within the samples, in order to understand the total metabolic differences. Orthogonal projections to latent structures discriminant analysis (OPLS-DA) was performed for the supervised multivariate statistical analysis using R packages. The significantly different metabolites were determined based on a combination of a statistically significant threshold of variable influence on projection (VIP) values obtained from the OPLS-DA model and two-tailed Student’s t tests (the *p* value) of the raw data. Metabolites with VIP > 1.0 and *p* < 0.05 were considered as significant. The variation in the quantitative information of the metabolites detected in each group was compared, and a graph was drawn for the first ten metabolites after Log_2_ treatment. The differential metabolites were submitted to the Kyoto Encyclopedia of Genes and Genomes database (KEGG, Kyoto Encyclopedia of Genes and Genomes, http://www.genome.jp/kegg/, accessed on 20 July 2021) for related pathway analysis. Python packages were used to identify statistically significantly enriched pathways using Fisher’s exact test. When the *p*-value of a metabolic pathway was less than 0.05, that pathway was considered to be statistically significantly enriched. Correlation matrixes between the gut bacterial species and gut microflora-related metabolites were generated using the Spearman correlation coefficient, as calculated using R software.

### 2.8. Statistical Analysis

T-test test, Wilcox rank sum test, and Tukey test were used to analyze whether the difference in species diversity between groups was significant. T-test and Wilcox rank sum test are performed when there are only 2 groups, and Tukey and Wilcox rank sum tests are performed when the group is greater than 2. The false discovery rate method was used for multiple test correction. *p* < 0.05 was considered significant.

## 3. Results

### 3.1. Diet Identification from Fecal Samples

**Figure 1:**
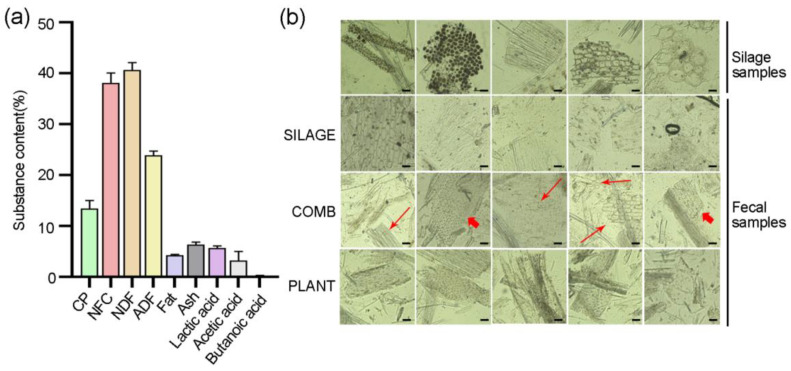
Analysis of silage contents and microscopic observations of silage and fecal samples. (**a**) Crude protein (CP), non-fiber carbohydrates (NFC), neutral detergent fiber (NDF), acid detergent fiber (ADF), fat, ash, lactic acid, acetic acid, and butanoic acid were determined in four batches of silage. (**b**) Microscopic images of cell types were applied to identify the fecal samples with different dietary regimes. Long arrowheads indicate the cell types that are same as the silage, and short arrowheads indicate the cell types that are same as the feces samples of PLANT diet. Bar = 100 μm.

We first analyzed the content of main components of the silage. The results showed that it mainly comprised 13.46% crude protein, 38.09% non-fiber carbohydrates, 40.64% neutral detergent fiber, 23.89% acid detergent fiber, 4.27% fat, 6.34% ash, 5.70% lactic acid, 3.23% acetic acid, and 0.18% butanoic acid (Figure 1a). In order to accurately identify the feces of Père David’s deer with different dietary regimes, the structure observation of the feces was further examined by microscopy (Figure 1b). Five types of plant cells were observed in the silage samples. These cells were also observed in the fecal samples of individuals that had been assumed to be feeding predominantly on silage (SILAGE diet), whereas none of these cell types was observed in the feces of individuals feeding on naturally occurring plants. There were special cell types in the PLANT diet, which were also observed in the feces from the COMB diet. However, the cell types of the feces from the COMB diet was similar to both the silage and natural plant feeding feces. Based on these observations, we could eliminate some of the undetermined samples, and then 15 fecal samples that showed a good relationship with the three dietary regimes (the SILAGE, COMB, and PLANT diets) were chosen for the following study.

### 3.2. Effect of Different Diets on the Diversity of the Gut Microbiota

**Figure 2:**
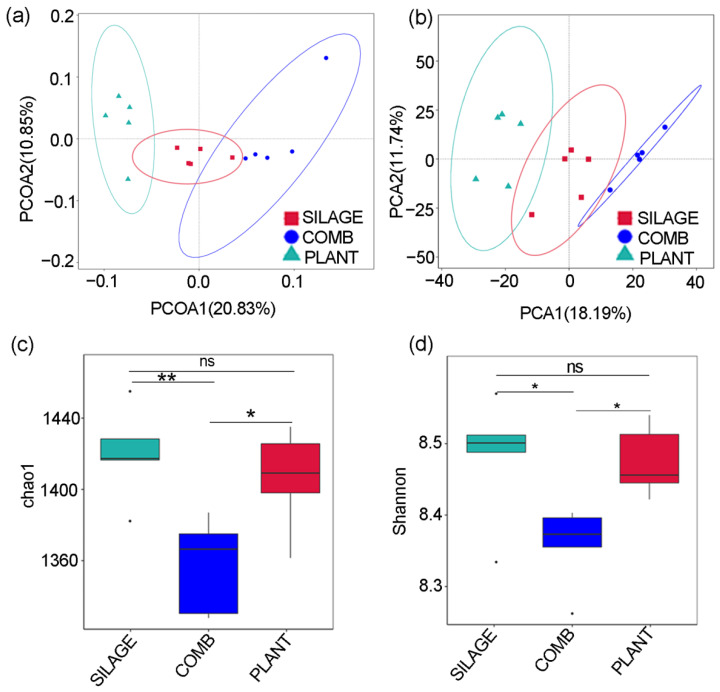
Diversity analysis of Père David’s deer fecal samples. Both the PCoA (**a**) and PCA (**b**) scatter plots of OTUs showed distinct clustering patterns for the PLANT, COMB, and SILAGE diets. Comparisons of alpha diversity, including the Chao1 index (**c**) and Shannon index (**d**), were conducted between the PLANT, COMB, and SILAGE diets. Data are presented as mean ± SEM (*n* = 5/group): * *p* < 0.05, ** *p* < 0.01, ns, no significant.

Both the PCoA and PCA analyses of the gut microbiome composition revealed that the deer eating the SILAGE, COMB, and PLANT diets were clearly different (Figure 2a,b). The microbial community richness indicated by the Chao1 estimators showed a significant decrease in the COMB group relative to the SILAGE group (*p* = 0.0059, Figure 2c). The community diversity estimated by the Shannon index was also consistently and significantly less in the COMB group relative to the SILAGE group (*p* = 0.0237, Figure 2d). However, the alpha diversity increased significantly in the PLANT group (Chao1, *p* = 0.0283; Shannon, *p* = 0.0305) compared with the COMB group. Furthermore, no significant differences in the alpha diversity were observed between the SILAGE and PLANT groups. Therefore, the alpha diversity of the gut microbiota was significantly lower in the COMB diet feces compared with those in the SILAGE and PLANT groups. 

### 3.3. Gut Microbiota Composition of Père David’s Deer Was Altered with the Different Dietary Regimes

**Figure 3:**
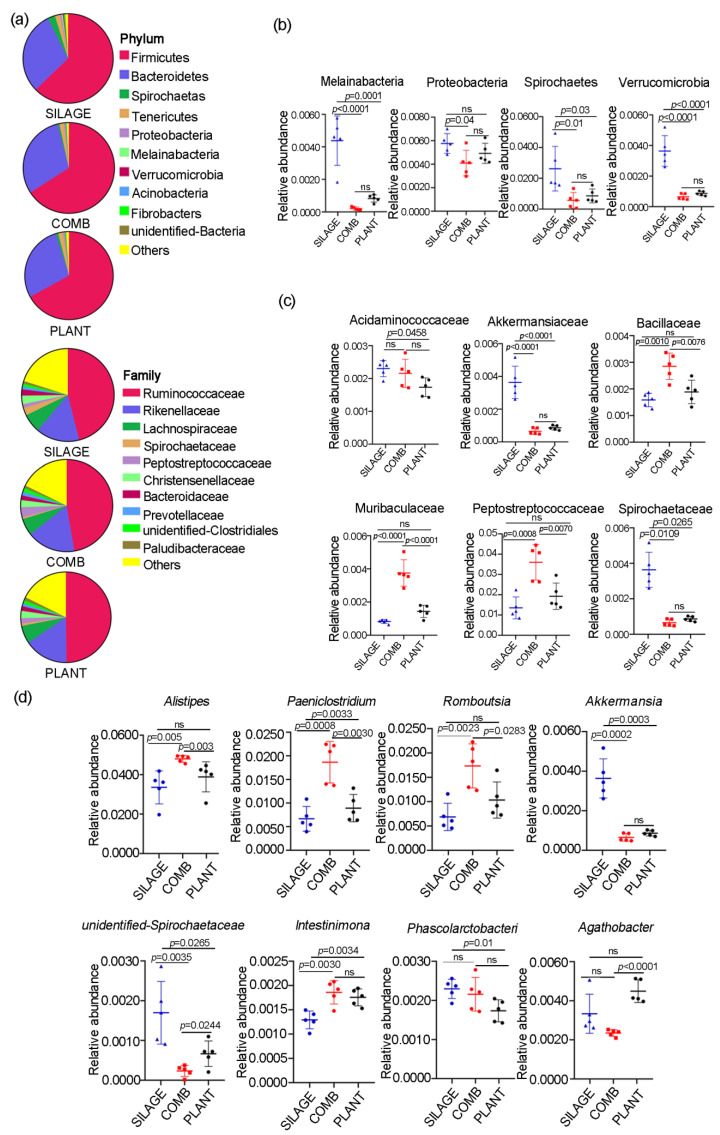
The abundance of gut microbiota of Père David’s deer under the different dietary regimes. (**a**) Bacterial phylum and family profiles in the gut microbiome for the PLANT, COMB, and SILAGE diets. (**b**) Comparisons of the relative abundance in the gut microbiome among the PLANT, COMB, and SILAGE diets at phylum level. (**c**) Comparisons between their relative abundance in the gut microbiome for the PLANT, COMB, and SILAGE diets at family level. (**d**) Comparisons of the relative abundance in the gut microbiome among the PLANT, COMB, and SILAGE diets at genus level. Data are presented as mean ± SEM (*n* = 5/group).

Approximately 16 bacterial phyla, 50 bacterial families, and 49 bacterial genus were identified in the three dietary groups (Figure 3a). Among these, two phyla (i.e., Firmicutes and Bacteroidetes) were the principal phyla in all of the samples analyzed, which accounted for more than a 90% abundance. However, there were no significant differences in each phyla among the three dietary regimes. A significant decrease in the relative abundance of four bacterial phyla (*Spirochaetes, Melainabacteria, Proteobacteria*, and *Verrucobacteria*) was found in the COMB group compared with the SILAGE group (Figure 3b), whereas the levels of these four phyla were significantly higher in the PLANT group compared with the COMB group. At the family level, there were no significant differences in each core family (i.e., *Ruminococcaceae, Rikenellaceae*, and *Lachnospiraceae*) among the three dietary regimes. However, the abundance of three taxa (i.e., *Bacillaceae, Muribaculaceae,* and *Peptostreptococcaceae*) was significant higher in the COMB group compared with the PLANT and SILAGE groups (Figure 3c), whereas *Akkermansiaceae* and *Spirochaetaceae* were less abundantly expressed in the COMB group and PLANT group compared with SILAGE group. At the genus level, compared with the SILAGE and PLANT groups, the abundance of *Alistipes*, *Paeniclostridium*, and *Romboutsia* was significant higher in the COMB group (Figure 3d). However, two genera, *Akkemansia* and *undentified-Spirochaetacease*, were significantly lower in both the COMB and PLANT groups compared with the SILAGE group. In addition, *Paeniclostridium* and *Intestinimonas* were significantly higher in the PLANT group. Therefore, the gut microbiota composition of Père David’s deer in the Dafeng Reserve was significantly different under these three dietary regimes, especially under the COMB diet. 

### 3.4. Fecal Metabolite Profiles Were Different under the Different Dietary Regimes 

**Figure 4:**
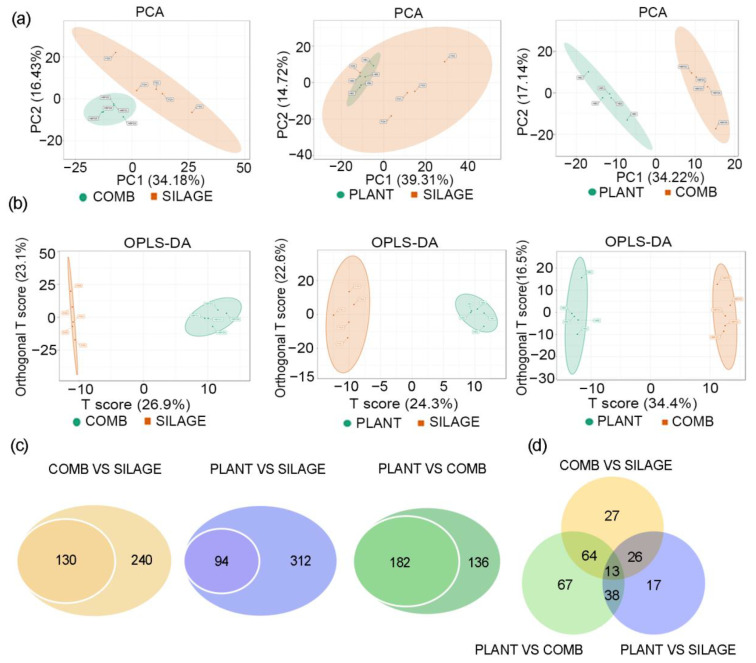
Comparisons of the total metabolite differences in fecal samples among the different dietary regimes. Scatter plots for the PCA results (**a**) and the OPLS-DA results (**b**) for the PLANT, COMB, and SILAGE diets. (**c**) Differential metabolites based on OPLS-DA analysis using VIP ≥ 1 and *p* < 0.05 as the filter for the COMB vs. the SILAGE diets, the PLANT vs. the SILAGE diets, and the PLANT vs. the COMB diets. (**d**) Venn diagrams showing the number of altered metabolites shared between the COMB vs. the SILAGE diets (orange), the PLANT vs. the SILAGE diets (blue), and the PLANT vs. the COMB diets (green).

Approximately 500 different metabolites were identified in all of the fecal samples. The PCA scatter plots showed clear separations between the SILAGE, COMB, and PLANT groups (Figure 4a). OPLS-DA models were constructed to classify the metabolite differences among these three groups. According to the OPLS-DA score plots based on the metabolic profile, almost all of the individuals could be clearly separated by their dietary regime (Figure 4b). In order to further identify the differential metabolites, results were screened using both the multivariate analysis with VIP > 1 and the univariate analysis with *p* < 0.05. Comparing the SILAGE group with the COMB group, the COMB group with the PLANT group, and the PLANT group with the SILAGE group by cross-comparisons, nearly 112, 96, and 92 differential metabolites were identified, respectively (Figure 4c). Among these, 13 differential metabolites were identified among all three groups, namely kynurenic acid, 3′-aenylic acid, DL-3,4-dihydroxyphenyl glycol, myoinositol, azelaic acid, β-nicotinamide mononucleotide, L-ascorbate, inosine, 6-methylnicotinamide, methionine sulfoxide, LysoPE 14:0, glycocholic acid, and N-acetylphenylalanine (Figure 4d). Overall, the different dietary regimes greatly affected the expression of fecal metabolites of Père David’s deer in the Dafeng Reserve. 

### 3.5. Differences of Fecal Metabolites under the Different Dietary Regimes

**Figure 5:**
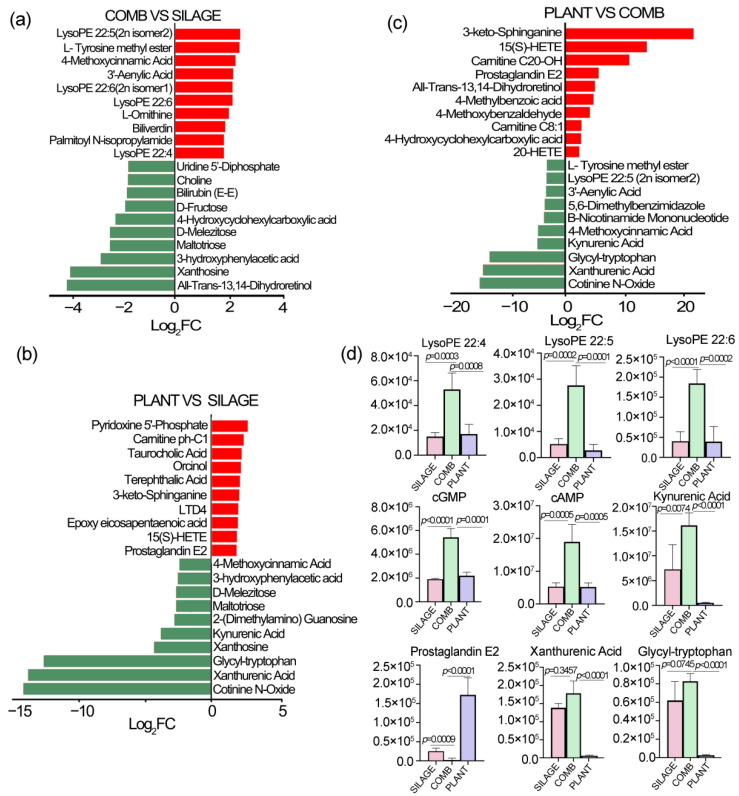
Significant differences in the fecal metabolites among the different dietary regimes. Log_2_FC of the top ten most distinguishing metabolites in the feces of Père David’s deer between the COMB and SILAGE diets (**a**), the PLANT and SILAGE diets (**b**), and the PLANT and COMB diets (**c**). (**d**) The abundance changes of nine different metabolites among three dietary regimes. Data are presented as mean ± SEM (*n* = 5/group).

The log_2_FC was processed in each group comparison, and the results of the top ten metabolites showing changes are displayed in Figure 5. Compared with the SILAGE group, several lyso-phosphatidylethanolamines (Lyso-PE), such as Lyso-PE (22:4, 22:6, 22:5), together with 4-methoxycinnamic acid, biliverdin, L-omithine, and 3′-aenylic acid, were significantly higher in the COMB group (Figure 5a). All-trans-13,14-dihydroretinol, 3-hydroxyphenylacetic acid, xanthine, maltotrios, D-melezitose, D-fructose, bilirubin (E-E), choline, and 5′UDP were significantly lower in the COMB group. Furthermore, different fecal metabolites, including pyridoxine, carnitine ph-C1, taurocholic acid, orcinol, LTD4, and prostaglandin E2, were significantly higher in the PLANT group. However, cotinine N-oxide, xanthurenic acid, glycyl-tryptophan, xanthosine, kynurenic acid, 2-(dimethylamino)-guanosine, maltotriose, and D-melezitos were significantly lower compared with the SILAGE group (Figure 5b). A comparison between the COMB and PLANT groups was also performed (Figure 5c). The results showed that 3-keto-sphinganine, carnitine C20-OH, prostaglandin E2, all-trans-13,14-dihydroretinol, and 20-HETE were significantly higher in the PLANT group. However, L-tyrosine methyl ester, cotinine N-oxide, xanthurenic acid, glycyl-tryptophan, kynurenic acid, lysoPE (22:5), 3’-aenylic acid, and β-nicotinamide mononucleotide were significantly lower compared with the COMB group. Based on these results, the changes in lyso-PE (including lysoPE 22:4, lysoPE 22:5, and lysoPE 22:6), the second-messenger molecules (cGMP and cAMP), tryptophan metabolites (including kynurenic acid, xanthurenic acid, and glycyl-tryptophan), and prostaglandin E2 were compared among the SILAGE, COMB, and PLANT groups (Figure 5d). The results showed that most of these metabolites were significantly higher in the COMB group compared with the SILAGE group. Thus, changes in dietary regimes led to corresponding changes in a large number of fecal metabolites of Père David’s deer in Dafeng Reserve.

### 3.6. Several Metabolic Pathways Were Different under the Different Dietary Regimes 

**Figure 6:**
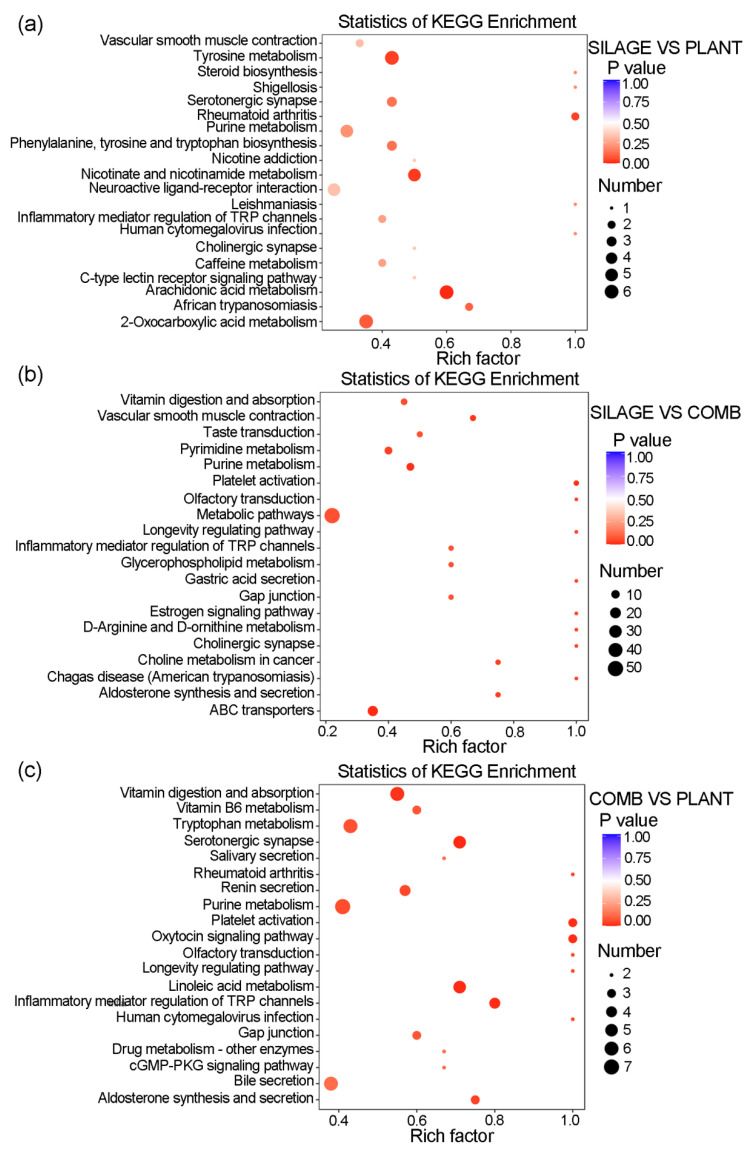
Changes in the metabolic pathways among the different dietary regimes of Père David’s deer. Bubble plots via KEGG enrichment analysis showing the top 20 most enriched metabolic pathways based on the distinguishing fecal metabolites of Père David’s deer between the PLANT and SILAGE diets (**a**), the COMB and SILAGE diets (**b**), and the PLANT and COMB diets (**c**). The rich factor is the ratio of the number of significantly different metabolites detected to the number of metabolites annotated in the pathway. The larger the value, the greater the degree of enrichment. The size of a dot represents the number of significantly different metabolites enriched in the corresponding pathway.

A KEGG enrichment analysis was performed to capture the changes in the metabolic pathways in Père David’s deer in the Dafeng Reserve during the transition from feeding on silage to naturally occurring plants. Many altered pathways were enriched, and the top 20 are listed in Figure 6. Several significantly altered metabolic pathways were identified between the SILAGE and PLANT groups, including tyrosine metabolism, phenylalanine, tyrosine and tryptophan biosynthesis, nicotinate and nicotinamide metabolism, neuroactive ligand–receptor interaction, arachidonic acid metabolism, 2-oxocarboxylic acid metabolism, serotonergic synapse, and purine metabolism (Figure 6a). Comparing the SILAGE and COMB groups, significantly changed pathways included vitamin digestion and absorption, vascular smooth muscle contraction, taste transduction, pyrimidine and purine metabolism, platelet activation, metabolic pathways, ABC transporters, inflammatory mediator regulation, glycerophospholipid metabolism, gastric acid secretion, gap junction, D-arginine and D-ornithine metabolism, and cholinergic synapse function (Figure 6b). Comparing the COMB and PLANT groups, significantly altered pathways included vitamin digestion and absorption, vitamin B6 metabolism, tryptophan metabolism, salivary secretion, purine metabolism, platelet activation, oxytocin signaling pathway, linoleic acid metabolism, gap junction, and bile secretion (Figure 6c). The digestion products and related metabolites of Père David’s deer in Dafeng Reserve changed corresponding to these changes in metabolic pathways according to the different dietary regimes. In the COMB group especially, more metabolites related to appetite, inflammation, vitamin metabolism, and amino acid metabolism were enriched in the corresponding pathways.

### 3.7. Different Gut Microbiota Related to the Metabolic Phenotype under the Different Dietary Regimes

**Figure 7:**
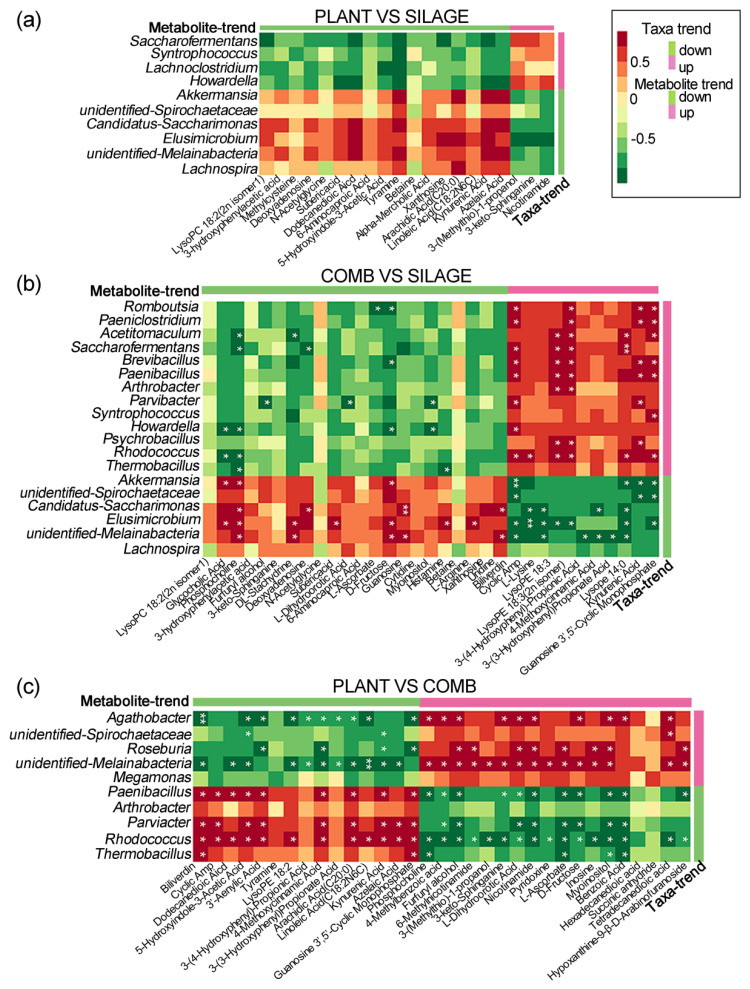
Functional correlations between the main genera and fecal metabolites among the different dietary regimes. Differential microbes and differential metabolites are based on Spearman correlation coefficients and are depicted as correlation heat maps comparing the PLANT and SILAGE diets (**a**), the COMB and SILAGE diets (**b**), and the PLANT and COMB diets (**c**). Red shows a positive correlation, and green a negative correlation. * *p* < 0.05, ** *p* < 0.01.

To explore the functional relationships among the altered gut microbiota, changed fecal metabolites, and different diets, three correlation matrices were formulated based on Spearman correlation coefficients. Comparing the SILAGE and PLANT groups, *Akkermansia*, *Candidatus-Saccharimonas*, and *unidentified-Melainabacteria* showed positive correlations with kynurenic acid and azelaic acid levels (Figure 7a). *Elusimicrobium* showed positive correlations with arachidic acid (C20:0), xanthosine, and dodecanedioic acid, but a negative correlation with nicotinamide, 3-keto-sphinganine, and 3-(methylthio)-1-propanol. Comparing the SILAGE and COMB groups, 19 gut microbiota displayed significant correlations with fecal metabolites at the genus level (*p* < 0.05, Figure 7b). For example, *Elusimicrobium* showed a significant correlation with 14 metabolites (*p* < 0.05), especially with cAMP (*p* < 0.001); *unidentified-Melainabacteria* showed a significant correlation with 11 metabolites (*p* < 0.05); *Candidatus-Saccharimonas* and *Rhodococcus* showed significant correlations with eight metabolites (*p* < 0.05); and *Akkermansia* showed a significant correlation with seven metabolites (*p* < 0.05). Comparing the PLANT and COMB groups, eight gut microbiota showed significant correlations with fecal metabolites at the genus level (*p* < 0.05, Figure 7c). Of these, *Rhodococcus* showed a significant correlation with 28 fecal metabolites; *unidentified-Melainabacteria* showed a significant correlation with 26 metabolites; *Parviacter* showed a significant correlation with 17 metabolites; and *Roseburia* showed a significant correlation with 12 metabolites. These data indicated that the COMB diet induced a reduction in the structure/composition of the gut microbiota in Père David’s deer, and substantially altered the fecal metabolic phenotype. 

## 4. Discussion

The gastrointestinal tracts of wild animals contain large, complex microbial communities, essential to the maintenance of the hosts’ health. Although a wild population of Père David’s deer has been successfully restored in the Dafeng Reserve, silage is still their main feed source in winter due to the limited amount and seasonal variation of edible plants in the reserve. From winter to summer, the dietary regime of most Père David’s deer in Dafeng Reserve changes from silage to a silage/plant combination, and finally to naturally occurring plants. Moreover, some individuals, even in summer or autumn, cannot easily find sufficient natural vegetation to meet their food requirements. The gut microbiota is important to maintain an animal’s physiological activities, nutrition, and health. Several studies have investigated the changes in the gut microbiota of captive and wild animals as their diets change [25,26,27]. To some degree, the COMB diet, which was the major dietary regime in spring, could be regarded as the transition from a silage to a natural vegetation dietary regime in Dafeng Reserve. However, there is no information relating to the effect of a combined diet of silage and natural vegetation on the gut microbiome of in Père David’s deer. Here, for the first time, we reveal the changes in the gut microbiome and metabolome of Père David’s deer with SILAGE, COMB, and PLANT diets. Microbial diversity is a useful bio-marker of the composition of the overall gut microbiota [28]. Several studies have demonstrated that gut microbial diversity is affected by captive conditions, some leading to a loss of microbial diversity [29,30]. In fact, diet was the major difference between captive and wild deer in the Dafeng Reserve. Sun et al. found that the alpha diversity in captive Père David’s deer was higher than in wild deer, but the differences were not significant [5]. Interestingly, we further revealed that the diversity was significantly lower in the COMB diet, in comparison with both the SILAGE and PLANT diets, suggesting that the natural plants intake may disrupt the balance of the gut microbiome. During the long winter in the Dafeng Reserve, the gut microbiota become well-adapted to the SILAGE diet. We deduce that the intake of the plant portion under the COMB diet inevitably shifts the balance of the gut microbiota of Père David’s deer. Additionally, the ratio of silage and natural plants is random and changes every day, which may lead to a constant disruption of the balance of gut microbiota. It seems that a steady diet (full SILAGE diet or full PLANT diet) is more beneficial for maintaining a high diversity of gut microbiota. Usually, a high bacterial diversity is associated with a better metabolic profile and high health status, whereas a loss in bacterial diversity is typically a feature of certain metabolic disorders [31]. Therefore, the obviously decreased alpha diversity in Père David’s deer feeding on the COMB diet demonstrated a reduced richness of the gut microbiota when animals fed on both silage and natural plants, which, perhaps, in turn, reduces the effectiveness of the gut microbiota.

Here, the gut microbiota richness of both major phylum (*Firmicutes* and *Bacteroidetes*) and families (*Ruminococcaceae*, *Rikenellaceae*, and *Lachnospiraceae*) was not significantly different under the three dietary regimes in Dafeng Reserve. However, significant differences in the amount of Firmicutes were reported in the intestines of deer from the Beijing Reserve and Shishou Reserve, which may be partly explained by greater variations in their food sources [12]. In our study, the abundance of some minority phylum and families was significant different in the COMB diet compared with the SILAGE diet. For example, the abundance of three phyla (*Spirochaetes, Melainabacteria*, and *Verrucobacteria*) and two families (*Akkermansiaceae* and *Spirochaetaceae*) was significantly lower under both the COMB diet and PLANT diet compared with the SILAGE diet. Moreover, at the genus level, the abundance of *Akkemansia* was also significantly lower in the COMB diet than in the SILAGE diet. 

*Akkermansia* was approximately 0.4% of the gut microbiota in Père David’s deer under the SILAGE diet, but was less than 0.1% under the COMB diet or PLANT diet. *Akkermansia*, an intestinal symbiont that colonizes the mucosal layer, is considered to be a next-generation beneficial microbe. Recent studies have revealed that *Akkermansia muciniphila* plays a key role in the maintenance of intestinal health, host metabolic modulation, and immune responses [32,33]. We therefore speculate that the SILAGE diet is beneficial in the maintenance of the intestinal level of *Akkermansia* in Père David’s deer. However, the COMB and PLANT diets decreased the levels of this beneficial microbe, which may be detrimental to the health of Père David’s deer in the Dafeng Reserve. Usually, there are several probiotics in the fermented silage, such as lactic acid bacteria, which are beneficial to digestion and animal health [34]. In the current study, detection of the effects of probiotics in silage has not been carried out, and further investigation is needed. Gut microbes contribute significantly to nutrient digestion and absorption, intestinal health, and immunity, and are essential for the survival and environmental adaptation of wild animals [35]. Accumulating evidence indicates that metabolites produced by gut microbes are crucial mediators of diet-induced host–microbial cross-talk, and that changes in them can cause differences in metabolic phenotypes [36,37]. The data presented here demonstrate that metabolite profiles can be clearly separated based on different dietary regimes. More metabolite profiles were significantly changed in Père David’s deer feeding on the COMB diet compared with the SILAGE or PLANT diets, indicating that the COMB diet affected the metabolic process in the digestive system more profoundly. The pathways affected include: taste transduction and gastric acid secretion, which may result from the influence of natural plant foods; vitamin digestion and absorption; base metabolism (pyrimidine and purine); and glycerophospholipid metabolism, which may result from the changes in food composition. Interestingly, platelet activation was also detected in individuals feeding on the COMB diet. We deduce that the intake of fresh plant material (rich in cellulose and pectin) might cause some damage to the intestinal mucosal cells of deer during dietary transition, and then might activate platelets to repair the damage. Another, more reasonable explanation may be that the imbalance of gut microbiota resulting from change in dietary regimes led to gut-derived lipopolysaccharide-induced platelet activation. Indeed, these two platelet activation pathways have been previously reported in human gut microbiota [38,39]. However, no platelet activation was detected in deer feeding on the PLANT diet, and this may be partly explained by their complete adaption to high-fiber plant food, or by the gut microbiota having adjusted to a steady state. The data also show that carbohydrate products (such as maltotriose, D-fructose, and D-melezitose), nucleotide products (xanthine, 5′dUDP), lipid products (choline and some short-chain fatty acid derivatives), and vitamin A were significantly more abundant in the SILAGE diet. Interestingly, several lysoPE (22:4, 22:6, and 22:5) levels were significantly higher in the COMB diet. LysoPE is a type of lysophospholipid. Previous studies have suggested that lysophospholipids are crucial for regulating epithelial integrity and physiological homeostasis [40]. A recent report showed that lysoPEs play an important role in sustaining the integrity of the intestinal epithelial barrier, and benefits health [41]. Here, the content of lysoPEs was significantly increased and glycerophospholipid metabolism was enhanced in the COMB diet. Additionally, the lysoPEs (14:0,18:3) were significantly correlated with several gut bacteria, such as *Saccharofermentans* and *Elusimicrobium*. One possible explanation is that, in response to the adverse effects of dietary changes in the intestinal epithelial gut barrier, several lysoPEs were produced by gut bacteria to regulate epithelial integrity and prevent the occurrence of inflammatory diseases. Wang et al. suggested that the gut microbes of Père David’s deer have potentially co-evolved with the host diet and reflect the local adaptation of translocated populations to new environments [13]. During the long winter in the Dafeng Reserve, the gut microbiota become well-adapted to the SILAGE diet [5]. When spring comes, the intake of the plant portion of the COMB diet inevitably shifts the balance of the gut microecology of Père David’s deer. As a herbivorous wild animal, the composition, abundance, and function of the gut microbes change in order to adapt to new dietary regimes. 

A recent analysis revealed that, in spring, the favorite food plants of Père David’s deer in Dafeng Reserve mainly included *Pennisetum alopecuroides*, *Spartina alterniflora Imperata cylindrica*, and *Phragmites communis* comprising crude fiber (24.06–34.88%), crude fat (0.89–1.64%), crude ash (8.28–14.89%), crude protein (5.25–9.76%), non-fiber carbohydrates (40.53–46.75%), and moisture (above 5.00%) [19]. We found that the silage used in the Dafeng Reserve can provide a steady nutritional resource, comprising (dry matter) crude protein (14.46%), non-fiber carbohydrates (38.09%,), neutral detergent fiber (40.64%), acid detergent fiber (23.88%), hemicellulose (16.76%), fat (4.27%), and ash (6.34%). Transitioning from the steady nutrient supply provided by silage to the variable nutritional content of different natural plants results in an imbalance in the intestinal microbe community and changes in the metabolite pathways in response to the increased intake of plant material, which may reduce the immunity of Père David’s deer in Dafeng Reserve, leading to a greater susceptibility to disease. In fact, diarrhea is frequent in early spring almost every year in deer in the Dafeng Reserve. In addition, gap junction disruption in the gut barrier increases gut permeability, the occurrence of diarrhea, and leaky gut syndrome [42]. The gap junction was significantly changed between the SILAGE diet and the COMB diet, indicating that the gut barrier may change during the transition from the SILAGE to the COMB dietary regimes. 

Although there were no significant differences in the diversity of the gut microbiota communities between the PLANT and SILAGE diets, significant differences in the abundance of gut microbes was observed in several phyla, families, orders, and genera. Moreover, kynurenic acid, xanthurenic acid, and glycyl-tryptophan levels were significantly higher under the SILAGE diet than the PLANT diet. Xanthurenic acid and kynurenic acid are the intermediate products of tryptophan metabolism. Consequently, the KEGG analysis showed an up-regulation of the tryptophan metabolism signaling pathway in the SILAGE group. Trp is an essential amino acid obtained from the diet, and its metabolism is now known to be a key modulator of the gut microbiota, impacting major physiological and pathological pathways [43]. In mammalian cells, Trp is primarily degraded through the kynurenine pathway (KP) (KP represents 95% of ingested Trp). A small amount of Trp (4–6%) is converted into tryptamine and indole metabolites by the gut microbiota [44]. Curiously, this does not result in a significant change in Trp metabolites. However, the PLANT diet improves the metabolic benefits of pyridoxine (vitamin B6), taurocholic acid, LTD4, prostaglandin E2, and nicotinamide (vitamin PP), together with the up-regulation of nicotinate and nicotinamide metabolism. In humans, the intestinal bacteria can provide 86% of the body’s vitamin B6 (pyridoxine) requirements [45]. Indeed, the significant positive correlations between vitamin B6 and *Rhodococcus* and between vitamin PP and *Rhodococcus*, *Parviacter*, and *Paenibacillus* were observed in the gut of Père David’s deer in Dafeng Reserve. This implies that a full PLANT diet could provide more vitamin B6 and vitamin PP than the COMB and SILAGE diets. Arachidonic acid is the precursor for the synthesis of prostaglandin, thromboxane, and leukotriene derivatives and plays an important role in the cardiovascular and immune systems of animals [46,47]. However, the PLANT diet has a relatively low starch content, resulting from significantly decreased quantities of maltotriose. All in all, compared with the SILAGE diet, the gut microbiota of Père David’s deer must change in order to adapt to the new PLANT dietary regime. We believe that the significantly enhanced tryptophan metabolism associated with the SILAGE diet may be more beneficial to the health of Père David’s deer in the Dafeng Reserve. On the whole, the SILAGE diet seems to be more nutritious for Père David’s deer in the Dafeng Reserve, but vitamin supplements should be considered in the future. 

Admittedly, our study has several limitations. First, the sample size (*n* = 5) is relatively small. Although 60 fecal samples were collected, only 15 samples were identified as representative samples. Considering the greater representation in Père David’s deer of Dafeng Reserve, a larger sample size will give more robust results. Second, the factors of sex and age were not considered in our study, which will also affect this result. Third, despite the convenience and non-invasiveness of fecal sampling, the microbiota data from feces do not fully represent that of the gut microbiota. Thus, using feces as a proxy to reveal the gut microbiota in Père David’s deer in this study may be not precise. Finally, the nutrient composition of the PLANT diet and COMB diet were not detected in our study, which limited us to explain the relationship between the food composition and gut microbes deeply.

## 5. Conclusions

For the first time, the current study revealed that the gut microbiome and fecal metabolome of individual Père David’s deer were profoundly different under the COMB diet, which may represent the dietary regime of most Père David’s deer in early spring. These differences included a lower diversity of the gut microbiota, more metabolic changes, an enhancement of adaptive metabolic pathways, and significant correlations between the gut microbiota and fecal metabolites, which may reduce the immunity of Père David’s deer feeding on the COMB diet. Generally, the COMB diet occurring during seasonal dietary transitions affects the nutrition and health of Père David’s deer greatly. Therefore, it is very important to improve the daily monitoring of Père David’s deer in semi-wild and wild areas during early spring. In addition, a wholly plant-based diet seems to result in inadequate tryptophan metabolism, suggesting that tryptophan supplementation should be considered. It is also necessary to add vitamin supplements (such as vitamin B6 and vitamin PP) to the SILAGE diet. These findings provide important information on which to base an improved management of the natural release of Père David’s deer into the Dafeng Reserve.

## Data Availability

All the data that support the findings of this study are available from the corresponding author. Raw sequencing data are deposited into the Sequence Read Archive (SRA; http://www.ncbi.nlm.nih.gov/Traces/sra/, accessed on 14 February 2022) of NCBI (SAR: PRJNA806748).
